# Albumin-to-fibrinogen ratio is an independent predictor of corticosteroid response and prognosis in patients with IgA nephropathy

**DOI:** 10.1186/s40001-023-01106-6

**Published:** 2023-04-03

**Authors:** Yu Zhang, Liping Man

**Affiliations:** Department of Nephrology, The Affiliated Taizhou People’s Hospital of Nanjing Medical University, Taizhou School of Clinical Medicine, Nanjing Medical University, No. 366 Taihu Road, Jiangsu 225300 Taizhou, China

**Keywords:** IgA nephropathy, Corticosteroid response, Prognosis, Albumin-to-fibrinogen ratio

## Abstract

**Background:**

The objective of this study was to investigate whether the albumin-to-fibrinogen ratio (AFR) can predict corticosteroid response and prognosis prediction among IgA nephropathy (IgAN) patients.

**Methods:**

Eligible participants with diagnosed IgAN who were scheduled to receive corticosteroid therapy for persistent proteinuria were recruited. Receiver operating characteristic (ROC) curve analysis was performed to evaluate the predictive value of AFR or estimated glomerular filtration rate (eGFR) for corticosteroid response in IgAN patients. Risk factors for corticosteroid response and prognosis were validated using univariate and multivariate Cox proportional analyses.

**Results:**

AFR and eGFR were both effective predictors of corticosteroid response in IgAN patients, with area under the curve (AUC) values of 0.686 and 0.643, respectively (*P* < 0.001 and *P* = 0.002). Baseline AFR level at biopsy was an independent risk factor for remission after corticosteroid therapy (HR: 2.38, 95% CI 1.32–4.07, *P* = 0.015), 50% decline in eGFR (HR: 0.78, 95% CI 0.69–0.89, *P* = 0.025), kidney failure (HR: 2.46, 95%CI 1.16–3.71, *P* = 0.016), and a composite event (HR: 2.13, 95%CI 1.28–3.34, *P* = 0.009) in IgAN patients.

**Conclusions:**

AFR level at biopsy was a potential predictor of corticosteroid response and prognosis among IgAN patients.

## Introduction

Immunoglobulin A nephropathy (IgAN) is the most common type of primary glomerular disease globally and is characterised by the predominant deposition of immune complexes containing IgA1 in the glomerular mesangium [1]. Clinical presentations of IgAN range widely, including asymptomatic microscopic haematuria and rapidly progressive kidney failure [1]. Within 10 years of IgAN onset, approximately 15–20% of patients will develop end-stage renal disease (ESRD) [2]. As a result, IgAN is a major cause of kidney failure among individuals with primary (glomerulonephritis) renal disease, who require replacement therapies [3]. Currently, angiotensin-converting enzyme inhibitors (ACEIs) and angiotensin receptor blockers (ARBs) are the most common therapeutic strategies for IgAN [4]. Steroid therapy is usually used for patients who have persistent proteinuria (over 1 g daily) and an estimated glomerular filtration rate (eGFR) over 50 mL/min/1.73 m [2] despite maximum conservative therapy [5]. If a patient has crescents with rapid progressive glomerulonephritis, he or she should be treated with a more intensive immunosuppressive regimen (for example, cyclophosphamide + steroids). Renal biopsy has been shown to play an essential role in the definitive diagnosis, disease progression evaluation, and prognosis prediction of IgAN patients [7]. However, the clinical efficacy of corticosteroids varies significantly among different patients. Moreover, few studies have examined predictive for the response to corticosteroids. Thus, it is necessary to identify potential predictive factors for corticosteroid response in IgAN patients.

Albumin is an effective biomarker for nutritional and inflammatory status evaluation, and fibrinogen is an essential protein in the coagulation cascade. The albumin-to-fibrinogen ratio (AFR), which combines these two biomarkers, has been used as a prognostic factor in malignancies, e.g. non-small-cell lung cancer [8] and advanced oesophageal cancer [9]. However, the associations among the AFR, corticosteroid response, and prognosis in IgAN patients remain unknown. Thus, this study attempted to investigate the predictive value of the AFR for corticosteroid response and prognosis in IgAN patients.

## Material and methods

### Patients

This was a single-centre, retrospective study with the approval of the Medical Institutional Ethics Committee of our hospital. This study was performed in accordance with the Declaration of Helsinki. Eligible participants with diagnosed IgAN admitted to the Department of Nephrology, Taizhou People’s Hospital, The Affiliated Hospital of Nanjing Medical University between June 2016 and December 2019 were recruited. The inclusion criteria were as follows: (a) East Asian populations aged over 18 years; (b) pathologically confirmed diagnosis of IgAN; (c) complete clinicopathological and laboratory data from medical records; (d) scheduled corticosteroid therapy for persistent proteinuria (> 1 g/24 h) after treatment with ACEIs or ARBs for 6 months; and (e) follow-up over 12 months. The exclusion criteria were as follows: (a) secondary IgAN; (b) presence of ESRD at admission; (c) conditions affecting albumin and fibrinogen levels, e.g. inflammation, infection, autoimmune disease, haematological disease, and liver dysfunction; (d) malignancies; and (e) no follow-up or complete data. Each enrolled participant signed an informed consent form.

The corticosteroid treatment strategy was performed according to the description by previous studies. In brief, methylprednisolone was administered via intravenous injection (1 g/d for three days) at 1, 3, and 5 months, and prednisone was administered orally (0.5 mg/kg/d, once every two days) for 6 months [10]. The enrolled patients were monitored monthly in the outpatient clinic. Proteinuria less than 1 g/24 h during follow-up was defined as disease remission in this study. Enrolled patients were followed up for as long as possible. For the primary observational endpoint (corticosteroid response), a 12-month follow-up was required (*n* = 245). For the second endpoint (50% decline in eGFR, ESRD, etc.), a 3-year follow-up was required (*n* = 189).

### Data collection

The clinicopathological data of the enrolled patients were extracted from the medical database. The demographics (age, sex), clinical data (blood pressure, history of macroscopic haematuria, and smoking habits), and comorbidities (diabetes, hypertension) were recorded. The level of eGFR at biopsy was calculated based on the Chronic Kidney Disease Epidemiology Collaboration (CKD-EPI) formula [11]. Two independent pathologists were invited to confirm histopathological classifications by Oxford classification methods [12, 13], with the contents of mesangial proliferation (M), segmental glomerulosclerosis (S), endocapillary hypercellularity (E), tubular atrophy/interstitial fibrosis (T), and crescents (C). Laboratory variables on the morning using fasting blood of the renal biopsy included haemoglobin, platelet count, white blood cell count, albumin, total cholesterol, fibrinogen, serum creatinine, uric acid, proteinuria, and eGFR (mL/min/1.73 m^2^).

### Prognosis definition

The primary observation endpoint was disease remission, which was defined above. A composite event of either a 50% decline in eGFR, ESRD, or death was set as the second observation endpoint. As reported by previous reports, ESRD was defined when renal replacement therapy was initiated or eGFR < 15 ml/min/1.73 m [14].

### Statistical analysis

Statistical analysis was performed using GraphPad Prism 8.0 (GraphPad Inc., San Diego, CA, USA) and SPSS 19.0 (SPSS Inc., Chicago, IL, USA). The Chi-square test, Student’s t test, or Mann–Whitney U test was used for data analysis as appropriate. The cut-off and predictive values of AFR and eGFR for corticosteroid response in IgAN patients were assessed by receiver operating characteristic (ROC) curve with the Youden index method. Risk factors for corticosteroid response and prognosis were validated using univariate and multivariate Cox proportional analyses. *P* < 0.05 was set as a significant difference.

## Results

### Patient characteristics

In total, 285 patients were initially recruited according to the inclusion criteria. Based on the exclusion criteria, 40 patients were excluded (5 with secondary IgAN, 6 with ESRD at admission, 13 with conditions affecting albumin and fibrinogen levels, 7 with malignancies, and 9 without complete data), and 245 patients were ultimately enrolled in the analysis. Of the 245 enrolled patients, one hundred fifty-nine achieved remission after corticosteroid therapy with a remission rate of 64.9% (159/245). The detailed clinicopathological characteristics associated with remission in IgAN patients are presented in Table 1. The mean age of the cohort was 38.8 years, and 52.2% (128/245) were male patients. Those patients in the remission group had a lower age (*P* = 0.003) and SBP at biopsy (*P* = 0.004) than those in the non-remission group. With respect to the Oxford classification, patients with an absence of M1 (*P* = 0.037), E1 (*P* = 0.023), and C1/C2 (*P* = 0.002) were more likely to achieve remission with corticosteroid therapy. No significant differences were observed in sex distribution, MAP, DBP, history of macroscopic haematuria, smoking habits, medications taken, comorbidities of diabetes, and hypertension between patients with or without remission (*P* > 0.05). In addition, patients with remission showed lower rates of 50% decline in eGFR (*P* = 0.009) and kidney failure (*P* = 0.015) within the 3-year follow-up.

**Table 1:** Clinicopathological variables associated with corticosteroid response in IgAN patients

Variables	Remission	Non-remission	*P* value
Number	159	86	–
Age (years)	37.7 ± 7.2	40.8 ± 8.4	0.003*
Gender, n (%)	–	–	0.803
Male	84(52.8)	44(51.2)	–
Female	75(47.2)	42(48.8)	–
MAP (mmHg)	95.4 ± 10.1	93.1 ± 11.2	0.103
SBP (mmHg)	126.6 ± 11.8	131.3 ± 12.1	0.004*
DBP (mmHg)	83.2 ± 9.9	82.7 ± 10.1	0.708
History of macroscopic haematuria, n (%)	19(11.9)	16(18.6)	0.155
Oxford classification, n (%)			
M1	44(27.8)	35(40.7)	0.037*
E1	39(24.5)	33(38.4)	0.023*
S1	79(49.7)	52(60.5)	0.106
T1-T2	43(27.0)	28(32.6)	0.364
C1-C2	56(35.2)	48(55.8)	0.002*
Current smoking, n (%)	8(5.0)	6(7.0)	0.531
Diabetes, n (%)	27(17.0)	16(18.6)	0.750
Hypertension, n (%)	43(27.0)	27(31.4)	0.472
Medications, n (%)	–	–	–
ACEI	16(10.1)	8(9.3)	0.848
ARB	128(80.5)	69(80.2)	0.959
SGLT2 inhibitor	53(33.3)	33(38.4)	0.430
3-year prognosis, n (%)	–	–	–
50% decline in eGFR	16/128 (12.5)	17/61 (27.9)	0.009*
Kidney failure	14/128 (10.9)	15/61 (24.6)	0.015*

P values were calculated by Student’s t test, Mann–Whitney U test, or Chi-squared test

*IgAN* Immunoglobulin A (IgA) nephropathy; *MAP* mean arterial blood pressure; SBP, systolic blood pressure; DBP, diastolic blood pressure; *ACEI* Angiotensin-Converting Enzyme Inhibitors; *ARB* Angiotensin Receptor Blockers; *SGLT-2* Sodium-dependent glucose transporters 2; *eGFR* estimated glomerular filtration rate

*P < 0.05

### Laboratory tests with corticosteroid response

Table 2 presents the levels of laboratory variables at biopsy. Patients who achieved remission with corticosteroid therapy had significantly higher eGFR and AFR than those without remission (*P* < 0.001). Moreover, patients with lower levels of uric acid and creatinine were associated with a better clinical response to corticosteroid therapy (*P* = 0.003 and 0.002, respectively). There were no significant differences in haemoglobin, platelets, white blood cells, total cholesterol, or proteinuria between patients with or without remission (*P* > 0.05).

**Table 2:** Laboratory tests associated with corticosteroid response in IgAN patients

Laboratory tests	Remission	Non-remission	*p* value
Number	159	86	–
Haemoglobin (g/L)	113.2 ± 8.2	111.5 ± 9.2	0.139
Platelet (10^9^/L)	177.3 ± 37.3	172.3 ± 34.7	0.306
White blood cell (10^9^/L)	7.0 ± 1.7	6.9 ± 1.6	0.654
Total cholesterol (mmol/L)	4.5 ± 0.7	4.6 ± 0.8	0.311
Uric acid (μmol/L)	355.4 ± 81.5	389.4 ± 91.3	0.003*
Serum creatinine (μmol/L)	77.3 ± 27.6	90.4 ± 35.4	0.002*
eGFR (mL/min/1.73 m^2^)	88.3 ± 20.1	78.1 ± 17.5	< 0.001*
Proteinuria (g/24 h)	3.1 ± 0.8	3.3 ± 1.1	0.104
AFR	7.9 ± 0.8	7.3 ± 0.9	< 0.001*

P values were calculated by Student’s t test or Mann–Whitney U test

*IgAN* Immunoglobulin A (IgA) nephropathy; *AFR* albumin-to-fibrinogen ratio; *UA* uric acid; *Scr* serum creatinine; *eGFR* estimated glomerular filtration rate

*P < 0.05

### AFR, eGFR, and corticosteroid response

**Fig. 1:**
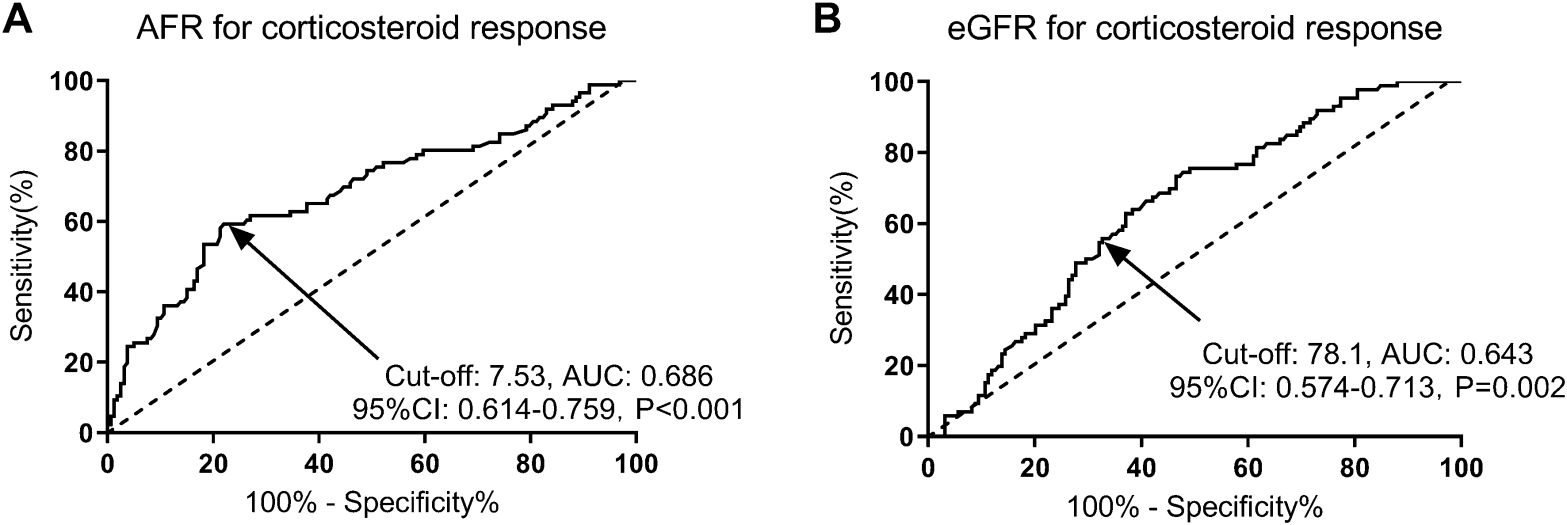
Predictive value of AFR (**A**) and eGFR (**B**) for corticosteroid response in IgAN patients by ROC curve. AFR and eGFR were both predictors of corticosteroid response in IgAN patients with AUC values of 0.686 and 0.643, cut-off values of 7.53 and 78.1, sensitivity values of 61.63% and 54.65%, and specificity values of 72.96% and 67.92%, respectively (*P* < 0.001 and *P* = 0.002). *AFR* albumin-to-fibrinogen ratio; eGFR, estimated glomerular filtration rate; *IgAN* Immunoglobulin A (IgA) nephropathy; *ROC* receiver operating characteristic; *AUC* the area under the curve; *CI* confidence interval

As illustrated in Fig. 1A, B, AFR and eGFR were both potential predictors of corticosteroid response in IgAN patients, with AUC values of 0.686 and 0.643, cut-off values of 7.53 and 78.1, sensitivity values of 61.63% and 54.65%, and specificity values of 72.96% and 67.92%, respectively (*P* < 0.001 and *P* = 0.002).

### Risk factors for corticosteroid response

As shown in Table 3, Oxford classification of C1/C2 (HR: 2.34, 95% CI 1.32–4.15, *P* = 0.018), eGFR (HR: 1.98, 95% CI 1.31–2.98, *P* = 0.012), and AFR (HR: 2.38, 95% CI 1.32–4.07, *P* = 0.015) were three independent risk factors for remission after corticosteroid therapy.

**Table 3:** Risk factors for corticosteroid response in IgAN patients by univariate and multiple Cox regression analyses

Variables	Univariate multivariate			
	HR(95%CI)	*p* value	HR(95%CI)	*p* value
Age (high vs low)	2.11(1.03–4.39)	0.041*	1.52(0.92–2.48)	0.088
SBP (high vs low)	1.73(0.86–3.49)	0.125		
History of macroscopic haematuria (yes vs no)	0.86(0.58–1.19)	0.308		
Oxford classification				
M0 vs M1	1.15(1.06–1.27)	0.009*	0.93(0.78–1.12)	0.446
E0 vs E1	1.21(0.51–2.71)	0.671		
C0 vs C1/C2	2.55(1.20–5.47)	0.011*	2.34(1.32–4.15)	0.018*
Uric acid (high vs low)	1.46(0.61–3.57)	0.377		
eGFR (≥ 78.1 vs < 78.1)	2.22(1.32–3.73)	0.003*	1.98(1.31–2.98)	0.012*
Serum creatinine (high vs low)	0.51(0.24–1.11)	0.094		
AFR (≥ 7.53 vs < 7.53)	3.63 (1.44–8.68)	0.005*	2.38(1.32–4.07)	0.015*

*SBP* systolic blood pressure; *IgAN* Immunoglobulin A (IgA) nephropathy; *AFR* albumin-to-fibrinogen ratio; *eGFR* estimated glomerular filtration rate; *HR* hazard ratio; *CI* confidence interval

^*^P < 0.05

### Prognostic factors for IgAN patients

**Fig. 2:**
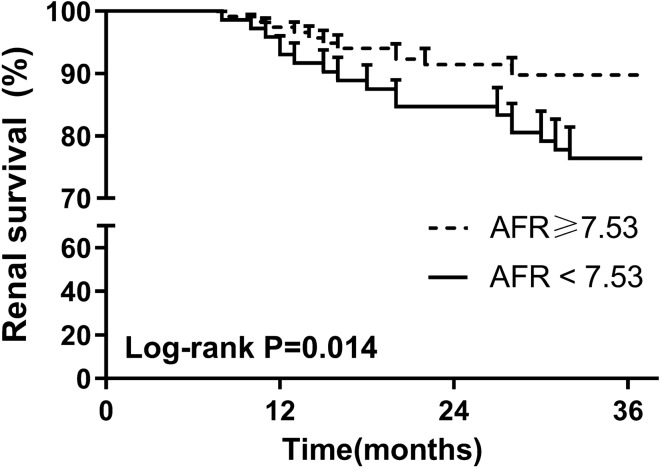
Association between AFR level and 3-year renal survival in IgAN patients by Kaplan‒Meier curve analysis. *AFR* albumin-to-fibrinogen ratio; *IgAN* Immunoglobulin A (IgA) nephropathy

**Fig. 3:**
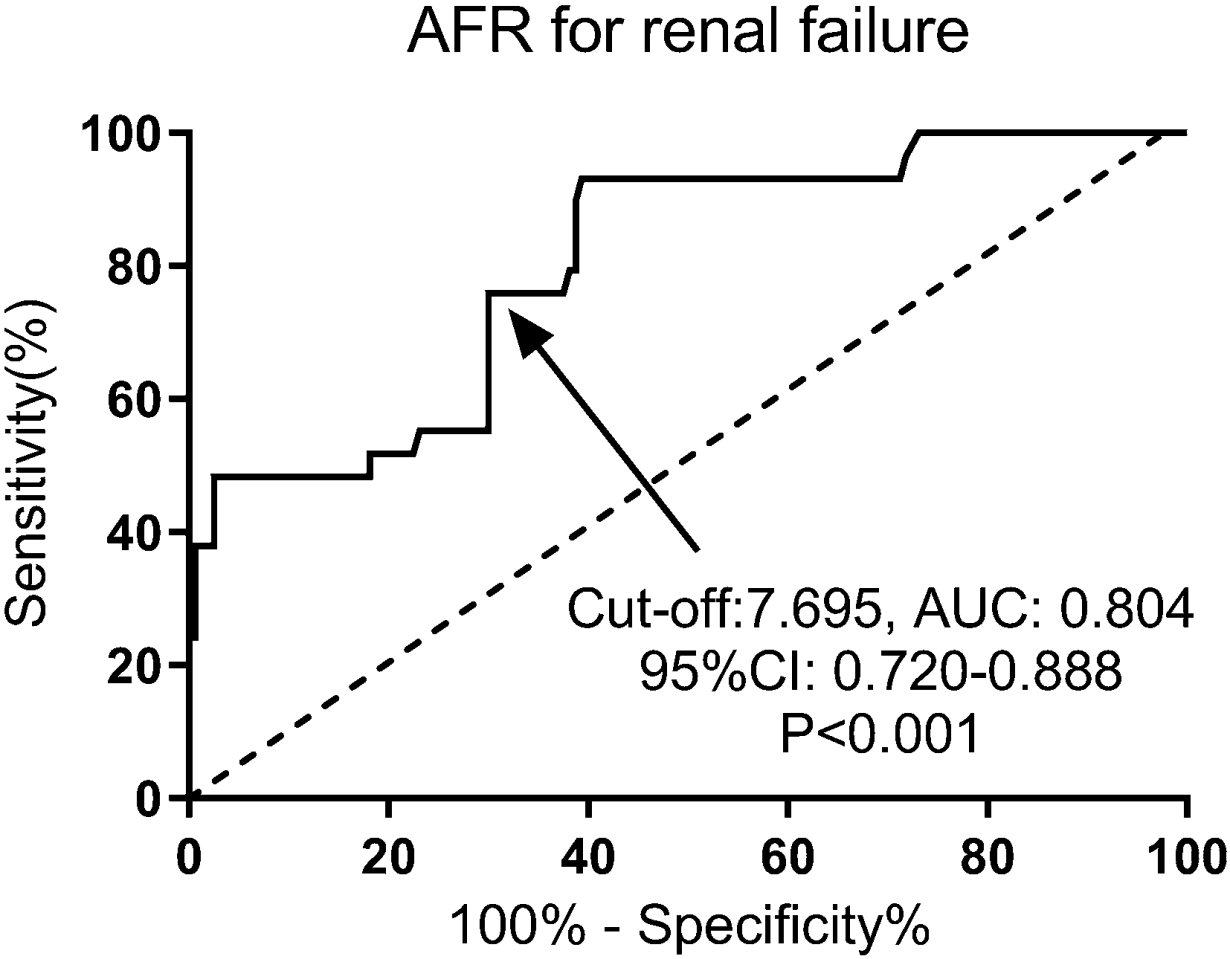
Predictive value of AFR for renal failure in IgAN patients by ROC curve analysis. *AFR* albumin-to-fibrinogen ratio; *IgAN* Immunoglobulin A (IgA) nephropathy; *ROC* receiver operating characteristic; *AUC* the area under the curve; *CI* confidence interval

Of all 245 enrolled patients, 189 had a follow-up over 3 years. Thus, the data of 189 patients were used for prognosis analysis. Taking a 50% decline in eGFR as an endpoint, eGFR (HR: 0.73, 95% CI 0.56–0.92, *P* = 0.013) and AFR (HR: 0.78, 95% CI 0.69–0.89, *P* = 0.025) were two prognostic factors for IgAN patients after corticosteroid therapy (Table 4). Interestingly, Oxford classification of M1 (HR: 1.83, 95% CI 1.06–3.12, *P* = 0.030) and AFR (HR: 2.46, 95% CI 1.16–3.71, *P* = 0.016) were two risk factors for kidney failure in IgAN patients (Table 5). Moreover, a composite event of either a 50% decline in eGFR, ESRD, or death was set as an observation endpoint. Our results showed that eGFR (HR: 1.76, 95% CI 1.09–2.54, *P* = 0.027) and AFR (HR: 2.13, 95% CI 1.28–3.34, *P* = 0.009) were two risk factors for a composite event in IgAN patients (Table 6). As shown by the Kaplan‒Meier renal survival curve in Fig. 2, patients with AFR≥7.53 were associated with a better 3-year renal survival (*P* = 0.014). In addition, AFR was a predictor of renal failure with a cut-off value of 7.695 and an AUC value of 0.804 (Fig. 3). All these results strongly indicated the prognostic role of AFR at biopsy in IgAN patients.

**Table 4:** Prognostic factors for 50% decline in eGFR in IgAN patients

Variables	Univariate multivariate			
	HR(95%CI)	*p* value	HR(95%CI)	*p* value
Oxford classification				
C0 vs C1/C2	0.88(0.80–0.97)	0.012*	0.96(0.90–1.03)	0.23
eGFR (≥ 78.1 vs < 78.1)	0.79(0.68–0.92)	0.008*	0.73(0.56–0.92)	0.013*
AFR (≥ 7.53 vs < 7.53)	0.71(0.51–0.98)	0.003*	0.78(0.69–0.89)	0.025*

*IgAN* Immunoglobulin A (IgA) nephropathy; *AFR* albumin-to-fibrinogen ratio; *eGFR* estimated glomerular filtration rate; *HR* hazard ratio; *CI* confidence interval

^*^P < 0.05

**Table 5:** Prognostic factors for kidney failure in IgAN patients

Variables	Univariate multivariate			
	HR(95%CI)	*p* value	HR(95%CI)	*p* value
Oxford classification				
M0 vs M1	3.01(1.90–4.89)	0.001*	1.83(1.06–3.12)	0.030*
eGFR (< 78.1 vs ≥ 78.1)	2.13(1.10–4.22)	0.021*	2.36(0.92–5.78)	0.072
Uric acid (high vs low)	3.98(2.79–5.72)	0.008*	1.12(0.72–1.72)	0.563
AFR (< 7.53 vs ≥ 7.53)	2.11(1.18–5.22)	0.009*	2.46(1.16–3.71)	0.016*

*IgAN* Immunoglobulin A (IgA) nephropathy; *AFR* albumin-to-fibrinogen ratio; *eGFR* estimated glomerular filtration rate; *HR* hazard ratio; *CI* confidence interval

^*^P < 0.05

**Table 6:** Prognostic factors for composite outcome in IgAN patients

Variables	Univariate multivariate			
	HR(95%CI)	*p* value	HR(95%CI)	*p* value
Age (high vs low)	2.04(1.03–3.67)	0.023*	1.69(0.88–2.89)	0.089
eGFR (< 78.1 vs ≥ 78.1)	1.91(1.24–2.94)	0.009*	1.76(1.09–2.54)	0.027*
AFR (< 7.53 vs ≥ 7.53 v)	2.41(1.33–4.23)	0.005*	2.13(1.28–3.34)	0.009*

*IgAN* Immunoglobulin A (IgA) nephropathy; *AFR* albumin-to-fibrinogen ratio; *eGFR* estimated glomerular filtration rate; *HR* hazard ratio; *CI* confidence interval

^*^P < 0.05

## Discussion

In this retrospective single-centre study, we demonstrated that the AFR level at kidney biopsy is an independent predictor of corticosteroid response and a strong prognostic factor for IgAN patients, as well as eGFR. Impaired eGFR levels at baseline are widely accepted as a traditional risk factor for kidney progression [15, 16]. In accordance with prior reports, we also confirmed that the eGFR at biopsy was a prognostic factor for kidney progression among IgAN patients. When comparing the predictive role for corticosteroid response, AFR had a higher predictive efficacy (a higher AUC, sensitivity, and specificity) than eGFR. Our results indicated AFR at biopsy as a potential parameter for corticosteroid response and prognosis in IgAN patients.

To date, there has been no consensus on the optimal therapy for IgAN. For patients with persistent proteinuria (> 1 g/day) and eGFR > 50 ml/min/1.73 m^2^, a 6-month course of corticosteroid, in addition to optimised supportive treatment, is strongly suggested by the Kidney Disease Improving Global Outcomes (KDIGO) Clinical Practice Guideline [17]. The application of corticosteroids along with ACEIs is reported to have a more effective antiproteinuric effect and a better prognosis than ACEIs alone [18]. Various studies have indicated various prognostic parameters of IgAN. For example, proteinuria, serum creatinine, and Oxford classification indices were considered risk factors for the early diagnosis of IgAN [19]. Moreover, some other new indicators also have excellent prognostic predictive ability in IgAN patients, e.g. serum IgG by Liu et al. [20], specific intercellular adhesion molecule-3 grabbing non-integrin by Wang et al. [21], and plasma acylcarnitines by Xia et al. [22]. However, studies regarding the potential indicators for corticosteroid response in IgAN patients are quite limited. A recent study by Yang et al. revealed the neutrophil-to-lymphocyte ratio (NLR) as a strong predictor of corticosteroid response in IgAN patients [2]. To the best of our knowledge, our study is the first to indicate AFR as an independent risk factor for remission achievement and prognosis in IgAN patients after corticosteroid therapy.

Serum albumin is acknowledged as a reliable biomarker of systemic inflammation and nutritional status [23]. Ni et al. have demonstrated that the time-averaged serum albumin expression independently predicts kidney progression in IgAN patients in remission after treatment [24]. Another study also identified hypoproteinaemia as an independent risk factor for an unfavourable renal outcome [25]. Furthermore, decreased serum albumin expression among IgAN patients was partially due to massive proteinuria, which is a significant factor associated with prognosis [26]. Fibrinogen, an essential protein in the coagulation cascade, has also been widely reported as a reliable prognostic factor in various diseases, e.g. localised renal cell carcinoma [27] and laryngeal squamous cell carcinoma [28]. Moreover, a recent case‒control study by Pan et al. indicated that serum fibrinogen expression is an independent risk factor for renal survival in primary IgAN patients [29]. AFR, taking albumin and fibrinogen together, has a more accurate clinical efficacy for prognosis prediction. A decreased AFR level at biopsy, which reflects the active systemic and glomerular inflammatory state and impaired nutritional status, might be possible explanations for the rapid decline in renal function and poor prognosis in IgAN patients.

Based on our results, we can confirm that risk stratification is necessary among IgAN patients. There have been many studies on treatment strategies in IgAN. For example, the Supportive Versus Immunosuppressive Therapy for the Treatment of Progressive IgA Nephropathy (STOP-IgAN) Trials in patients with IgAN and substantial proteinuria indicated that immunosuppression added to supportive care had no beneficial effect on renal function over three years [30–32]. In addition, clinical trials of target drugs for IgAN are currently ongoing, including LNP023 (complement Factor B inhibitor), atacicept, and RC-18 (recombinant human BLyS receptor antibody fusion proteins that target both BAFF and APRIL) [33].

This study has some limitations. First, this was a retrospective study performed in a single-centre institution with a relatively small cohort sample size. Second, the duration of follow-up was relatively short. Third, whether the modulation of AFR levels (e.g. albumin supplementation) could improve the corticosteroid response and prognosis remains unknown. Moreover, a composite event of a 50% decline in eGFR was used in this study; however, it is usually suggested for clinical trials instead of retrospective studies. It is better to use eGFR < 15 ml/min/1.73 m^2^, dialysis, and kidney transplantation. Forth, patient demographic difference is a potential confounder in treatment success of IgAN. Finally, the predictive value of AFR (AUC, sensitivity, and specificity) for corticosteroid response is relatively low.

In conclusion, the AFR level at biopsy was a potential predictor of corticosteroid response and prognosis among IgAN patients.

## Data Availability

All data generated or analysed during this study are included in this published article.
